# Mechanical Properties of Natural-Fiber-Reinforced Biobased Epoxy Resins Manufactured by Resin Infusion Process

**DOI:** 10.3390/polym12122841

**Published:** 2020-11-29

**Authors:** Mauricio Torres-Arellano, Victoria Renteria-Rodríguez, Edgar Franco-Urquiza

**Affiliations:** 1 National Council for Science and Technology (CONACYT—CIDESI), National Center for Aeronautic Technologies (CENTA), Carretera Estatal 200, km 23, Querétaro 76265, Mexico; mauricio.torres@cidesi.edu.mx; 2 Center for Engineering and Industrial Development (CIDESI), Graduate Department, Av. Playa Pie de la Cuesta 702, Querétaro 76125, Mexico; ana.renteria@cidesi.edu.mx

**Keywords:** natural fibers, biobased resins, biolaminates, mechanical properties

## Abstract

This work deals with the manufacture and mechanical characterization of natural-fiber-reinforced biobased epoxy resins. Biolaminates are attractive to various industries because they are low-density, biodegradable, and lightweight materials. Natural fibers such as Ixtle, Henequen, and Jute were used as reinforcing fabrics for two biobased epoxy resins from Sicomin^®^. The manufacture of the biolaminates was carried out through the vacuum-assisted resin infusion process. The mechanical characterization revealed the Jute biolaminates present the highest stiffness and strength, whereas the Henequen biolaminates show high strain values. The rigid and semirigid biolaminates obtained in this work could drive new applications targeting industries that require lightweight and low-cost sustainable composites.

## 1. Introduction

Fiber-reinforced polymers (FRPs), also known as laminated composites, are composite materials made of a polymer matrix reinforced with unidirectional long fibers or woven fabrics. The fibers are usually carbon, glass, or aramid. The primary function of the fibers is to carry loads and provide the composite with enough stiffness. The matrix confines the reinforcements, maintaining the structure and distributing the loads to protect the fibers from environmental conditions. The epoxy resins exhibit excellent adhesion and high compatibility with carbon fiber. However, they are expensive and considered neurotoxic and carcinogenic due to one of their precursors: bisphenol-A. Synthetic fibers such as fiberglass are toxic, require chemical treatment, and certainly a high quantity of energy for their production. Furthermore, they represent a health risk for workers when proper precautions are not followed [1,2,3,4,5].

One alternative to solve these severe disadvantages is to develop biolaminated composites, which are ecological composite materials that consist of natural fibers and epoxidized vegetable oil resins, which restrict the use of bisphenol-A [4,5,6,7]. There is extensive literature on the use of natural plant fibers as reinforcement for thermosetting resins. There are various types of natural plant fibers in Mexico, the most common of which come from the agave family (Asparagaceae/Agavaceae): Ixtle (Agave vivipara) and Henequen (Agave fourcroyes). The latter is the fiber used mainly to manufacture textiles due to its rigidity and ease of weaving. In the last years, they have attracted attention as innovative reinforcements of polymer composites oriented to nonstructural automotive parts and aircraft cabin interiors.

It is well documented that green composites’ mechanical properties improve with modified plant fibers because chemical treatments can favor the compatibility with the polymer matrix. For example, Ali et al. [8] investigated the effect of fluorocarbon, hydrocarbon, and hybrid fluorocarbon on the mechanical properties and moisture regain of Jute composites. For hybrid fluorocarbon, the composites contained treated Jute fibers that showed better properties because of the improved fiber–matrix interface. Going further with chemical treatments, Koyuncu et al. [9] studied the alkaline treatment by sodium hydroxide (NaOH) on cotton composites’ mechanical properties. The DMA results obtained revealed higher storage moduli and glass transition temperature (T_g_), attributed to the fiber treatment improving the cohesion between fiber and matrix. Other authors have investigated nanofillers’ use to improve the mechanical properties of composites reinforced with natural fibers. Rehman et al. [10] investigated the effect of microcrystalline cellulose (MCC) particles and alkaline treatment on the tensile, bending, and impact properties of Jute/bioepoxy composite, improving the tensile parameter by at least 48% when adding 7 wt.% of MCC. Jabbar et al. [11] reported the creep behavior of green composites by varying the weight fractions of pulverized Jute modified with alkali, and the DMA results showed a considerable increase in the viscoelastic parameters by increasing the Jute content.

Working with natural materials to prepare biolaminates has been challenging due to the lack of comprehensive property data for plant fibers. The absence of reliable data limits the utilization of natural, plant-based fibers in composite products. Moreover, thermoset resins with a biobased carbon content of approximately 40–60% can be unstable and very reactive under some pressure and temperature conditions. Different processes can epoxidize biobased epoxy resins from vegetable oil (commonly soybean seeds), such as the Prileschajew method [12].

The lack of specific information about the development and preparation of biolaminate composites forces us to look for suitable manufacturing alternatives for these materials [13,14,15]. The manufacturing process plays a huge role in the mechanical performance of biolaminates [16,17]. The hand lay-up process is an open molding method suitable for making a wide variety of biolaminates, and several research works [8,9,10] describe the manufacture of Jute/epoxy biolaminates using this process. However, the disadvantages of hand lay-up are (i) nonconstant fiber volume, (ii) health issues such as causing allergy or inhalation of volatiles, and (iii) low reproducibility. Some alternatives, such as bulk molding compound (BMC), a bulky mixture of chopped fibers, resin paste, and fillers, have been recently investigated. Puglia et al. [18] wrote a review concerning the treatment, manufacturing process, and opportunities of natural fibers for automotive applications. More recently, Sreenivasan et al. [19] discussed gel time, specific gravity, and volume fraction of kenaf/polyester bulk composites. They found that the specific gravity is lower as the fiber loading and fiber length increase. Despite these procedures’ advances, they are not suitable for fabrics or long natural fiber composites. From this perspective, vacuum-assisted resin infusion (VARI) seems to be the most suitable process to produce long natural fiber composites for diverse applications [17,20,21].

Some advantages of the VARI process includes (i) low investment cost: typically a little more investment than that required to make composite parts using hand lay-up in open molds; (ii) medium-volume fiber composites: capable of producing minimum weight, suitable-performance laminates with properties comparable to those of laminates produced by resin transfer molding (RTM) or compression molding; (iii) clean process: VARI allows the reinforcement materials to be handled dry, which reduces the workforce exposure to the resin; and (iv) healthy assurance: allergic reactions to resins such as epoxy can be avoided [20,21]. Studies on the use of the VARI process to prepare natural-fiber-reinforced synthetic thermoset resins are numerous. For example, Hossain et al. [22] proposed Jute-composite analysis regarding different stacking sequences. They evaluated the mechanical properties in the tensile and three-point bending tests, observing that as the fiber volume was 25%, these composites could be used for nonstructural applications, such as construction, furniture, and commodities. Another example is the experimental characterization of a Jute/epoxy–cork sandwich under impact and indentation. Hachemane et al. [23] evaluated the impact of energy and cork density on sandwich plate damage behaviors. They used the VARI process to manufacture composites with Jute–cork as a reinforcement. They concluded that as resin infiltrates the agglomerated cork’s pores, a difference in density (160 to 310 kg/m^3^) leads to a change in the local material stiffness. However, there is a lack of information reporting the mechanical properties of different natural-fiber-reinforced bioepoxy resins with different biomass contents, and this is the gap this work intends to fill.

With the described state-of-the art, the objective of this investigation is to characterize the mechanical properties of biolaminated composites based on Mexican and Asian endemic plant fibers impregnated with biobased epoxy resins and manufactured using the VARI process. The goal is to keep promoting the benefits of these biolaminates in industries with massive production of sustainable composite materials.

## 2. Experimental

### 2.1. Materials

**Figure 1:**
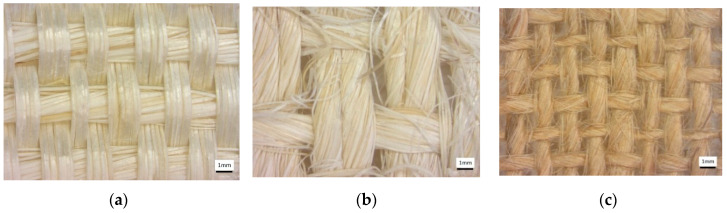
Photographs corresponding to natural fabrics: (**a**) Ixtle, (**b**) Henequen, and (**c**) Jute.

Three natural fibers, Ixtle and Henequen (belonging to the Asparagus family) from Mexico and Jute fiber (Corchorus olitorius) from Asia, were downselected for this study (Figure 1).

Ixtle and Henequen yarn fibers with thread counts of 30 and 35 were purchased to Cordeleria Santa Ines, Yucatan, Mexico. The yarn’s average length was 2000 m and was rolled on a reel for the following weaving steps. Both natural fibers were woven in a plain weave configuration by Mexican artisans from Queretaro’s state in Mexico. Henequen and Ixtle fibers have an elastic modulus of 13 and 27 GPa, a tensile strength of 13 and 18 MPa (ASTM C1557), and a density of 1.12−and 1.02 g/cm^3^ (ASTM D3822), respectively. According to the supplier, mechanical extraction is the process used to extract the Henequen fibers, and it promotes long fibers with a rough surface, and the maceration process is used to extract the Ixtle fibers and conducts to a smooth surface.

**Figure 2:**
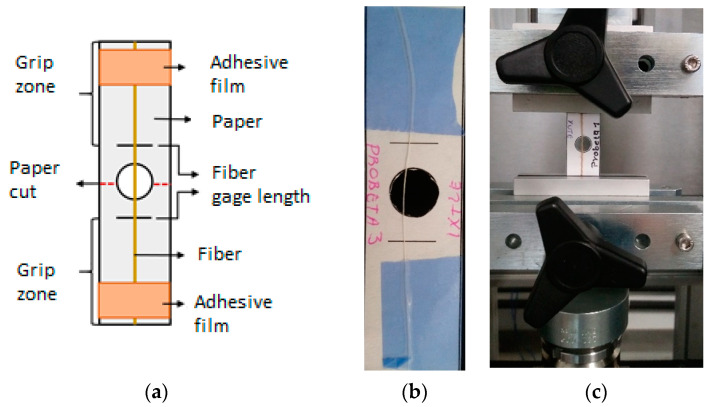
Experimental test setup for natural fibers: (**a**) schematic representation of the specimen, (**b**) Jute specimen, and (**c**) tensile test setup.

Bangladesh Jute fiber of 12 mm yarn with a thread count of 70 threads was imported by Bolsas Publicitarias, Cuernavaca, Morelos, Mexico in a plain wave configuration. Jute fibers have an elastic modulus of 67 GPa, a tensile strength of 45 MPa (ASTM C1557), and a density of 1.23 g/cm^3^ (ASTM D3822). It is well documented that cellulose influences plant fibers’ mechanical properties, hemicellulose is related to biodegradability, and lignin provides thermostability to the fibers [24]. Table 1 presents more technical data obtained from experimental results previously performed in our laboratory and literature. We calculated the mechanical properties of natural plant fibers used in this work using one thread from the natural-fiber yarns. The specimen test setup is shown in Figure 2. We tested three different gauge lengths of fibers of 20, 30, and 40 mm at room temperature (23 °C ± 1 °C) and a crosshead speed of 2 mm/min in an MTS Series 647 universal testing machine (MTS Systems Corporation, Eden Prairie, MN, USA) equipped with hydraulic wedge grips and a load cell of 1 kN. The experimental test comprised five repetitions for each gauge length.

In this work, we used two commercial biobased epoxy resins to impregnate the plant fibers through the VARI process to manufacture the biolaminated composites. The epoxidized vegetable oil (EVO) Surf Clear, with a biobased carbon content of approximately 40%, and the SD EVO fast hardener from Sicomin Epoxy Systems^®^ (Châteauneuf les Martigues, France) was mixed in a ratio by volume of 2/1. The Sicomin Resin SR GreenPoxy 56 (labeled in this work as GP) is an epoxy resin that has up to 56% of its molecular structure coming from plant origin. This resin was mixed with the SZ 8525 hardener from Sicomin Epoxy Systems^®^ in a volume ratio of 100/32. According to the supplier, EVO and GP biobased resins were extracted from vegetable oils, with no access to their chemical structure, offering an alternative due to the combination of specific attributes such as low viscosity, biodegradability, and high epoxy functionality. Table 2 lists the mechanical properties of both bioresins according to the technical datasheet.

### 2.2. Manufacturing of Biolaminates

**Figure 3:**
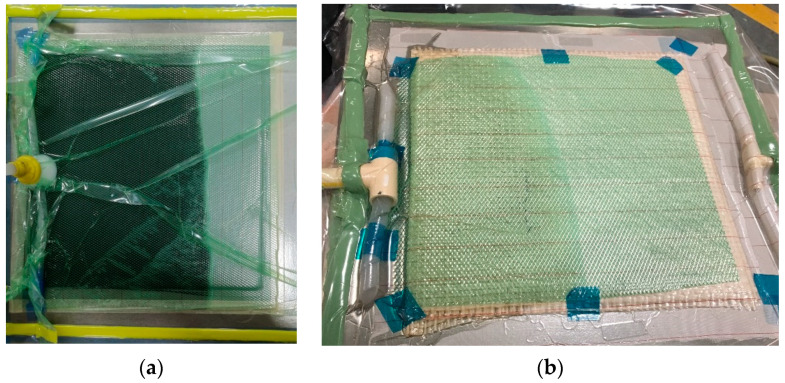
Photographs of manufacturing infusion impregnation process corresponding to: (**a**) vacuum-assisted resin infusion (VARI) with the epoxidized vegetable oil (EVO) biobased epoxy resin, (**b**) VARI with the Sicomin Resin SR GreenPoxy 56 (GP) biobased resin.

We used the VARI process to manufacture biolaminates. The vacuum pressure was −20 inHg, and the curing reaction lasted 24 h at 25 °C. Biolaminates have two plain weave layers, with a [0]_2_ stacking and 300 mm × 300 mm × 2.5 mm as nominal laminate dimensions. On the top of the substrate, peel ply and distribution mesh were placed over the fiber, and the entire configuration was covered with a vacuum bag and carried out using sealant tape. After air evacuation, the resin was infused at room temperature. The resin inlet and outlet positions were at the edges of the biolaminates, respectively. Figure 3 shows the schematics of the VARI process during the impregnation of natural fibers for manufacturing biolaminates.

**Figure 4:**
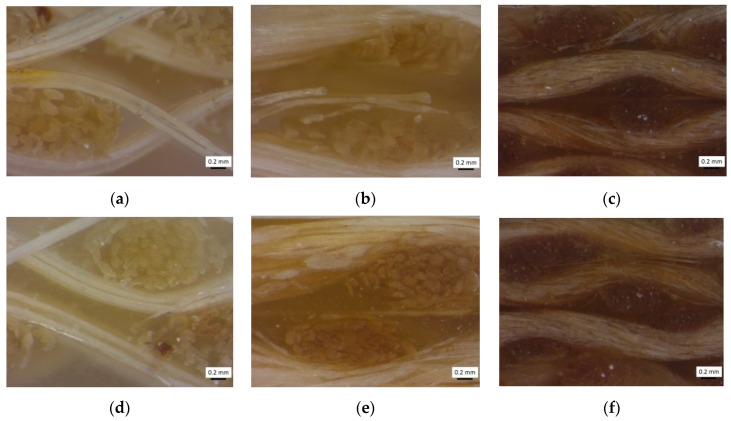
Optical micrographs corresponding to the biolaminates: (**a**) Ixt/EVO, (**b**) Hen/EVO, (**c**) Jute/EVO, (**d**) Ixt/GP, (**e**) Ixt/ GP, and (**f**) Ixt/ GP.

Figure 4 presents the optical micrographs obtained from the cross-section of the thickness of each of the biolaminates. The samples were cut into random areas and polished for observation at different magnifications. All samples revealed similar microstructural features, which indicates repeatability in the manufacturing process. The optical micrographs were taken by employing a stereoscopic ZEISS (SteREO Discovery.V8, Oberkochen, Germany).

It is possible to appreciate similar features between the Ixtle and Henequen fibers, as expected, although the Ixtle is more defined than the Henequen fiber because of the extraction process, as appreciated in Figure 1. Yute fabrics are composed of thin threads that promote the fibers closer to each other with relatively higher packing than the agave fiber composites. In all cases, the presence of microvoids was detected, and fabric distortions were not observed.

The stiffness and strength of the biolaminates can be improved by increasing the volume fraction of the fibers. Fiber and void volume fractions are essential parameters in evaluating the quality of composite materials. However, popular standard methods, such as pyrolysis or acid digestion, are not possible to follow in green composites because of the natural fibers. Therefore, in this work, the volume fraction of each constituent (fiber and bioresin) was calculated using the following equation:

(1) $$v_{i}=m_{i}\frac{\rho_{c}}{\rho_{i}}$$

where $v_{i}$ is the volume fraction of each constituent (fiber and bioresin), $m_{i}$ is the mass fraction (g), $\rho_{i}$ and $\rho_{c}$ are the densities (g/cm^3^) of each constituent and composite, respectively. Table 3 presents the calculated volume fractions. In this work, the bioresin densities were calculated following the ASTM D792—20. The values obtained were 1.178 g/cm^3^ for EVO and 1.253 g/cm^3^ for GP resin. The natural fiber densities were obtained following the ASTM D3822, and employing an analytical balance Mettler Toledo (XPE504, Columbus, OH, USA). The values are presented in Table 1.

### 2.3. Mechanical Testing for Biolaminates

The mechanical parameters of the biolaminates were determined from the unidirectional tensile tests and flexural bending. The specimens were cut using a diamond sawing of 1 mm width. For tension, specimens with nominal dimensions of 250 × 25 × 2.5 mm and a gauge length of 150 mm were tested according to the ASTM D3039-3039M. For bending, specimens with nominal dimensions of 80 × 12.5 × 2.5 mm were tested according to the ASTM D7264. Procedure A outlines a three-point loading system for center loading. In this work, the specimen lies on a support span, with a 16:1 span-to-thickness ratio, and the load is applied to the center by the loading nose producing three-point bending [29].

**Figure 5:**
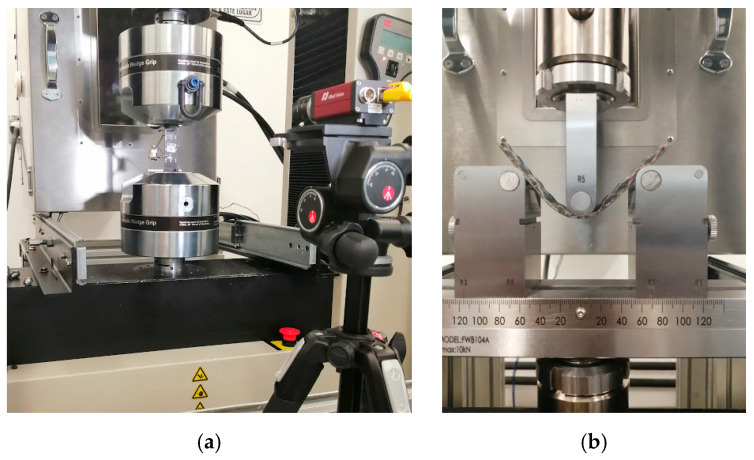
Photographs of mechanical testing setup for biolaminates corresponding to: (**a**) tensile test with the videoextensometer; (**b**) bending test.

A universal testing machine Instron (647, Cerdanyola, Barcelona, Spain) with a loading capacity of 100 kN, was used. The experimental tests were conducted under displacement control at a crosshead speed of 0.5”/min (1.27 mm/min) until the failure of the specimen. The elongation was recorded for the tensile tests using the noncontact videoextensometer MTS (Advantage Video Extensometer AVX, MTS Systems Corporation, Eden Prairie, MN, USA) with a 25 mm lens. The videoextensometer recognizes patterns on surfaces to acquire measurement data for strain calculations processed by MTS TestSuite™. The set up for both tensile and bending tests are depicted in Figure 5.

## 3. Results and Discussion

### 3.1. Tensile Properties of Biolaminates 

**Figure 6:**
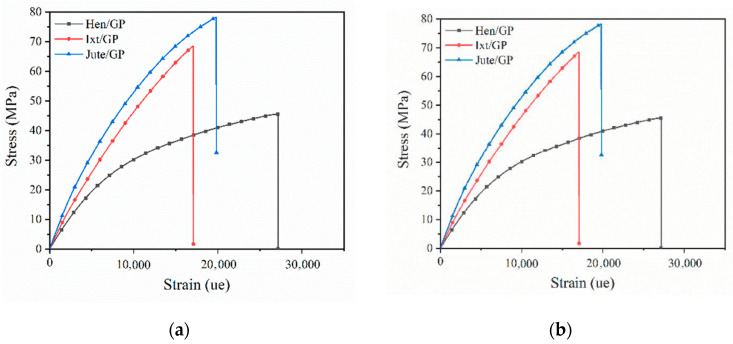
Representative tensile stress vs. strain curves for the natural-fiber-reinforced biobased resins: (**a**) biolaminates with the EVO resin; (**b**) biolaminates with the GP resin.

Figure 6 shows the representative stress—strain curves of biolaminates.

For all curves, two regions are identified. The first region exhibits a linear elastic behavior, from the beginning of the test until reaching 3000 microstrains. Hereafter, the second region shows a nonlinear behavior due to the typical sticking–slipping mechanism of woven laminates. In this second region, progressive elastoplastic behavior occurs before sudden failure.

**Figure 7:**
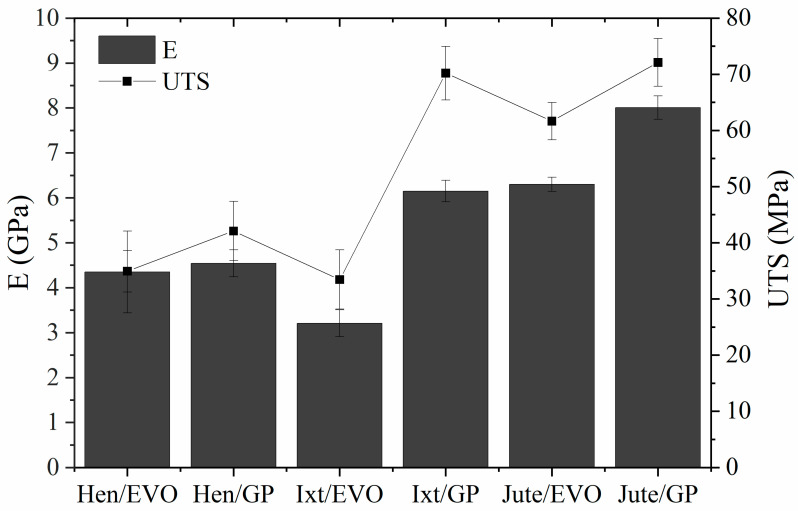
Tensile properties of the EVO and GP biolaminates.

The mechanical parameters, such as the Young’s modulus (E) and an ultimate tensile strength (UTS) obtained for the EVO and GP biolaminates reinforced with natural fibers, are compared in Figure 7. We add commercial fiberglass (GFRP) data (biaxial 3 K woven glass fiber and Q2 epoxy resin by Quintum, Mexico City, Mexico) for comparison purposes. The GFRP has a Young’s modulus of 27 GPa, a UTS of 337 MPa, a flexural modulus of 17 GPa, and an ultimate flexural strength of 273 MPa.

The results reveal that the tensile properties of biolaminates containing GP bioresin are in general higher than those obtained with the EVO biobased resin, which is reasonable if we consider the GP biobased resin has better compatibility with natural fibers because it contains up to 56% of its molecular structure coming from plant origin. The most notorious differences were found in the Ixt/GP biolaminates whose modulus, strength, and maximum load values are double those obtained with the EVO resin, although the strain is slightly lower (Figure 6). The results could be associated with the better compatibility between natural fibers and GP bioresin, which favor the interface bonding of these biolaminates and improve their mechanical properties.

With respect to composites with the EVO bioresin, the Jute/EVO biolaminate exhibits higher mechanical performance than the biocomposites reinforced with Henequen and Ixtle. The stiffness of Jute/EVO was approximately 97% and 45% higher than the Ixtle and Henequen biolaminates, respectively. Moreover, the strength and maximum load of Jute/EVO were higher up to 80, and over 100% with respect to the other two biocomposites. It seems the cellulose content of natural fiber favors the modulus and fiber volume of the biolaminates, referred to in Table 1 and Table 3, respectively. The strength is notoriously higher with Jute fiber, whereas the Ixtle and Henequen biocomposites are similar if the standard deviation is considered. In this case, the differences in strength could be related to the intrinsic properties of the natural fibers used in this work. However, the process of extraction could play a relevant role because Henequen is obtained by a mechanical process that promotes better adhesion than Ixtle fiber, which is extracted by maceration process that reduces the adhesion to EVO, giving similar strength results to the Hen/EVO biolaminate and the lowest values of strain.

Regarding composites using the GP bioresin, the differences in tensile properties related to the intrinsic properties of the natural fiber did not show significant trends. Hen/GP was the biolaminate with the lowest tensile parameters, whereas the strength and maximum load of the Jute/GP and Ixt/GP biocomposites were similar, and Jute/GP was the biolaminate with significant stiffness. The results suggest that the Ixtle and Jute fibers are more compatible with the GP bioresin than Henequen and provide better stress transfer mechanisms. Various parameters influence the mechanical properties of fiber-reinforced composites including the fiber orientations, fiber–matrix adhesion, stress transfer at the interface, and fraction volume of fiber density. Jute/GP biolaminates combine the best parameters to obtain high tensile mechanical properties: (i) high fabric closure factor (porous and gaps between yarns are small), (ii) high fiber volume fraction, (iii) low-viscosity resin with good impregnation, and (iv) good wettability, resulting in a remarkable bonding interface between the fiber and resin.

Comparing biolaminates with commercial GFRP, we found the following insights. For the Young modulus, on the one hand, EVO biolaminates have 1/9, 1/6, and 1/4 for Ixtle, Henequen and Jute, respectively. On the other hand, GP biolaminates have 1/4, 1/6, and 1/3 for Ixtle, Henequen and Jute, respectively. For UTS, the performance is lower as the EVO biolaminates have 1/10, 1/10, and 1/5 for Ixtle, Henequen, and Jute, respectively, and the GP biolaminates have 1/5, 1/8, and 1/5 for Ixtle, Henequen, and Jute, respectively. In summary, the Jute/GP biolaminates, per their nature highlighted before, show a better ratio for nonstructural applications where GFRP are used.

### 3.2. Bending Properties of Biolaminates 

**Figure 8:**
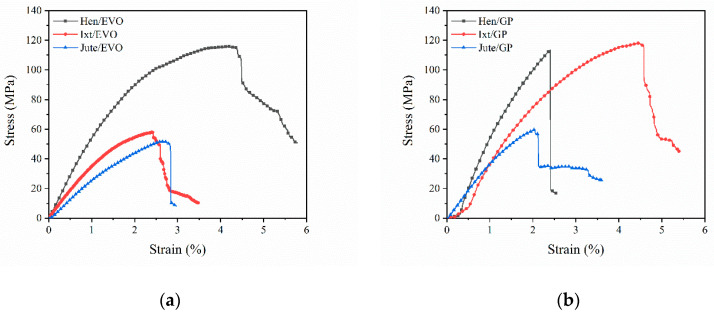
Representative bending stress vs. strain curves for the natural-fiber-reinforced biobased resins: (**a**) biolaminates with the EVO resin, (**b**) biolaminates with the GP resin.

**Figure 9:**
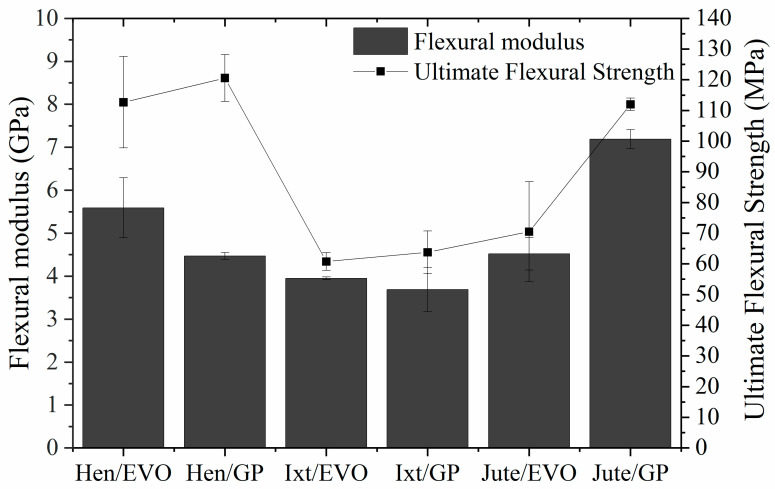
Bending properties of the EVO and GP biolaminates.

The representative stress–strain curves for the Ixtle, Henequen, and Jute fibers impregnated with bioepoxy resins EVO and GP under bending are presented in Figure 8. The bending parameters, such as flexural modulus and ultimate flexural strength, are compared in Figure 9.

For all specimens, two regions were perfectly identified. The first region exhibits a linear elastic behavior followed by a nonlinear behavior due to the epoxy resin’s elastoplastic nature, the interlaminar shear strength (ILSS), and the sliding of woven tows. Progressive damage before sudden failure was possible to observe.

Flexural properties exhibit higher values compared to the tensile properties. However, in contrast to the tensile configuration, the bending properties do not seem to follow any tendency regarding biobased resins’ biomass content. Henequen- and Ixtle-fiber-reinforced EVO have a higher flexural modulus than GP biolaminates, but the flexural strength reveals opposite behavior. Nonetheless, Jute/GP has a flexural modulus and bending strength notoriously higher than the Jute/EVO biolaminates, which may be explained by the improved interfacial adhesion between the matrix and fiber fabrics.

On the other hand, analyzing the results obtained with the EVO bioresin makes it easy to identify that Hen/EVO has better mechanical performance in bending. Its flexural modulus is 37% and 19% higher than the Ixtle and Jute biolaminates, respectively, and the bending strength was higher by up to 68%. It is necessary to highlight the ultimate bending strain of the biocomposites reinforced with Jute and Ixtle is quite similar, whereas Hen/EVO presents large ultimate strains (≈5.8%). As previously mentioned, the EVO bioresin does not seem to provide high compatibility with natural fibers. Therefore, the bending configuration results could be related to the cellulose content and the fraction volume of biolaminates.

Regarding the composites using the GP bioresin, Jute/GP exhibits the highest flexural modulus, followed by Henequen and Ixtle biocomposites. The flexural modulus difference can be at least 60% and 95% compared with Henequen and Ixtle, respectively. On the other hand, Hen/GP presents the highest strength with the larger strain (≈6%), whereas the Jute and Ixtle biolaminates fail at quite similar strain values. The fiber–matrix interface plays an essential role in determining the mechanical properties of composite materials. The mechanical results indicate the Jute fiber is the most suitable to reinforce biobased resins because of the excellent bonding between Jute fabrics and the biobased epoxy matrix. However, it is essential to consider the effect of different volume fractions of fiber on the flexural strength and modulus for various fiber fabric types of composites. Increasing the volume fraction of fiber on the biocomposites will result in a significant increase of flexural stiffness, as tensile and impact strengths are increased by the addition of natural fibers. Furthermore, Jute fabrics have a higher density, followed by Henequen and Ixtle, which could influence the flexural modulus. However, under bending conditions, Henequen fabrics promote higher flexural strength values.

Comparing the biolaminates with commercial GFRP, we found the following insights. For flexural modulus, on the one hand, EVO biolaminates have 2/9, 5/16, and 1/4 for Ixtle, Henequen, and Jute, respectively. On the other hand, GP biolaminates have 1/5, 1/4, and 2/5 for Ixtle, Henequen, and Jute, respectively. For flexural strength, EVO biolaminates have 2/9, 2/5, and 1/4 for Ixtle, Henequen, and Jute, respectively, and the GP biolaminates have 2/9, 4/9, and 2/5 for Ixtle, Henequen, and Jute, respectively. The flexural strain of biolaminates is 2, 3.6, and 0.3 times higher than GFRP for Ixtle, Henequen, and Jute, regardless of the bioresin employed. Biolaminates present a more flexible behavior, which leads them to applications where an excellent strength–flexibility ratio is needed. Once again, the Jute/GP biolaminates show a better ratio for applications where comparable GFRP properties are needed.

### 3.3. Discussion of Overall Mechanical Performance of Biolaminates

Jute–epoxy laminate systems have good properties, competing with random mat glass-reinforced plastics, wood, and wood plastics [30]. In this study, Jute fiber exhibited higher properties due to two main reasons: (1) low tow diameter and (2) high closure factor (the low gap between tows). These two factors enhance the stiffness and strength of the Jute biolaminate [13,31]. Additionally, the excellent interaction between Jute and biobased epoxy resins has improved the overall mechanical properties compared with values reported in the last decade [32].

For the Ixtle biolaminates, there is a strong dependence of the mechanical properties on the bonding interface between fibers and resin, as most agave fibers tow disorderly arranged microfibrils, which provide a larger surface for bonding. Therefore, surface treatment of these kinds of fibers is suitable to improve the adherence between fibers and resin [33,34]. As cited above, the molecular compatibility with plant-like-based resins is an essential factor for manufacturing biolaminates with Ixtle fibers. Additionally, few studies have shown that Ixtle (sisal family fibers) and Jute composites have shown similar behavior after modifying their fiber surface [3,35].

The Henequen biolaminates fit very well with large elongations and low-strength applications [35]. Henequen biocomposites could afford potential applications, which request composites with a high fiber volume (more than 35%). As agave plants (sisal) have gained attention in the last decade, Henequen seems to be a good fit for sustainable composites if additional fiber surface treatment is pursued to increase tensile, compression, and impact strengths [34]. As stated before, yarn count, fiber nature, and artisanal weaving affect the mechanical properties of the studied biolaminates. This study is the first approach to characterize Mexican raw natural fibers, which are weaved by hand. Of course, if an industrial weaving approach is developed, the fabrics’ variability will decrease, and constant mechanical properties would be expected. Some examples of instant applications for the studied biolaminates can be plastic and wood furniture, isolation panels/walls, docks panels, and beams, or even repairing wood-based dock piles.

### 3.4. Failure Evaluation of Tensile Specimens

At the end of each tensile test, a failure examination is necessary to analyze the fracture surface, infer the operative fracture mechanisms, and classify their primary failure mode. All failure modes are according to the specific codes of ASTM D3039.

**Figure 10:**
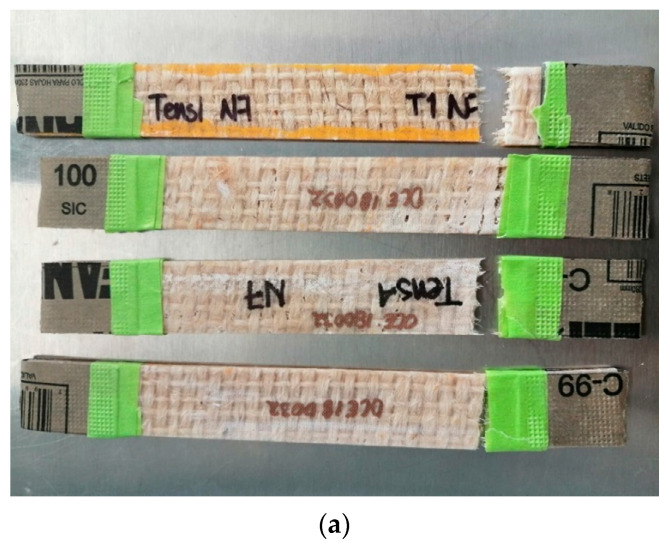

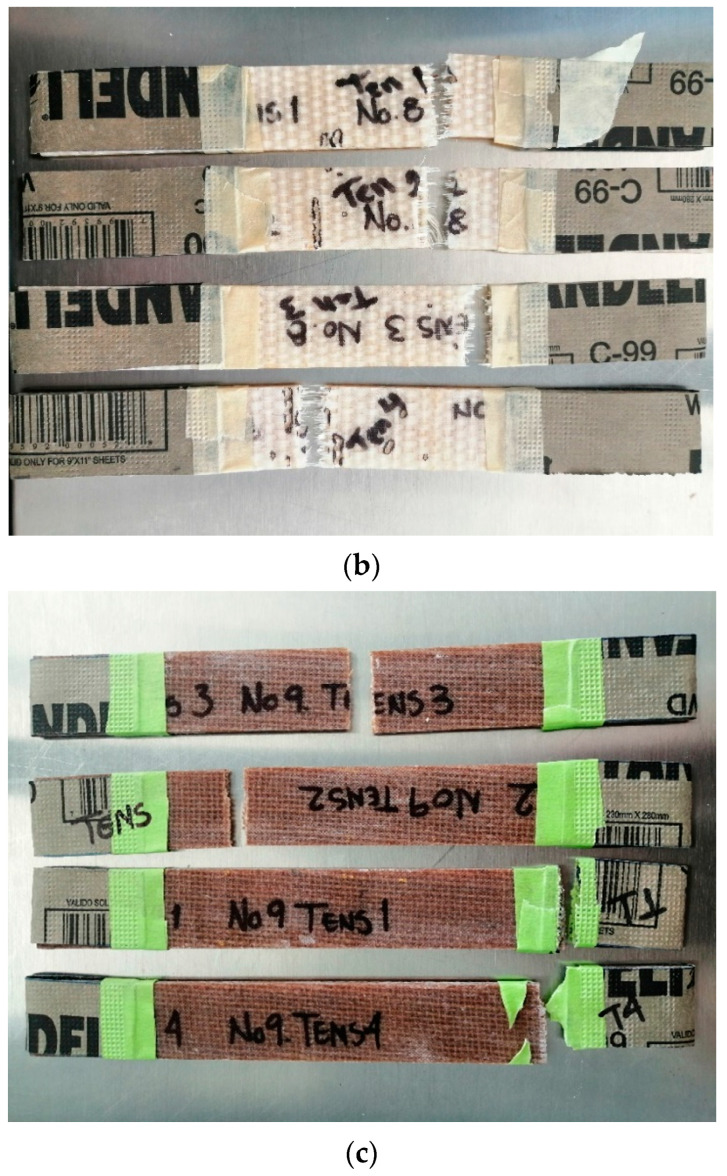
Photographs corresponding to the failure modes in tension for: (**a**) Hen/EVO, (**b**) Ixt/EVO, (**c**) Jute/EVO biolaminates.

In the first instance, for the natural-fiber-reinforced EVO biolaminates (Figure 10), the failure modes are as follows. The Hen/EVO biolaminates all fail by sudden fracture type LAT (Lateral, At grip/Tab, Top), as shown in Figure 10a. It is possible that grip force, together with one of the laminate faces’ roughness, has a strong influence on the Hen/EVO failure mode. Additionally, warp and weft do not have symmetrical tow sizes; hence, the mechanical behavior depends on fabric morphology. For the Ixt/EVO biolaminates, most fail by sudden fracture type LGM (Lateral, Gage, Middle) and present type LGT (Lateral, Gage, Top), as shown in Figure 10b. As the Ixtle fabric has a tighter tow gap and a more equilibrated tow size at the warp and weft, failure is expected in the gage zone. Finally, the Jute/EVO biolaminates all fail by a different fracture type: LAT (Lateral, At grip/Tab, Top), LGM (Lateral, Gage; Middle), and LGT (Lateral, Gage; Top), as shown in Figure 10c. As Jute has the high closure factor and the smallest tow diameter, ergo, fabric distortion, and fiber waviness play an essential role in the fracture mechanisms involved, numerous for a single type of laminate.

**Figure 11:**
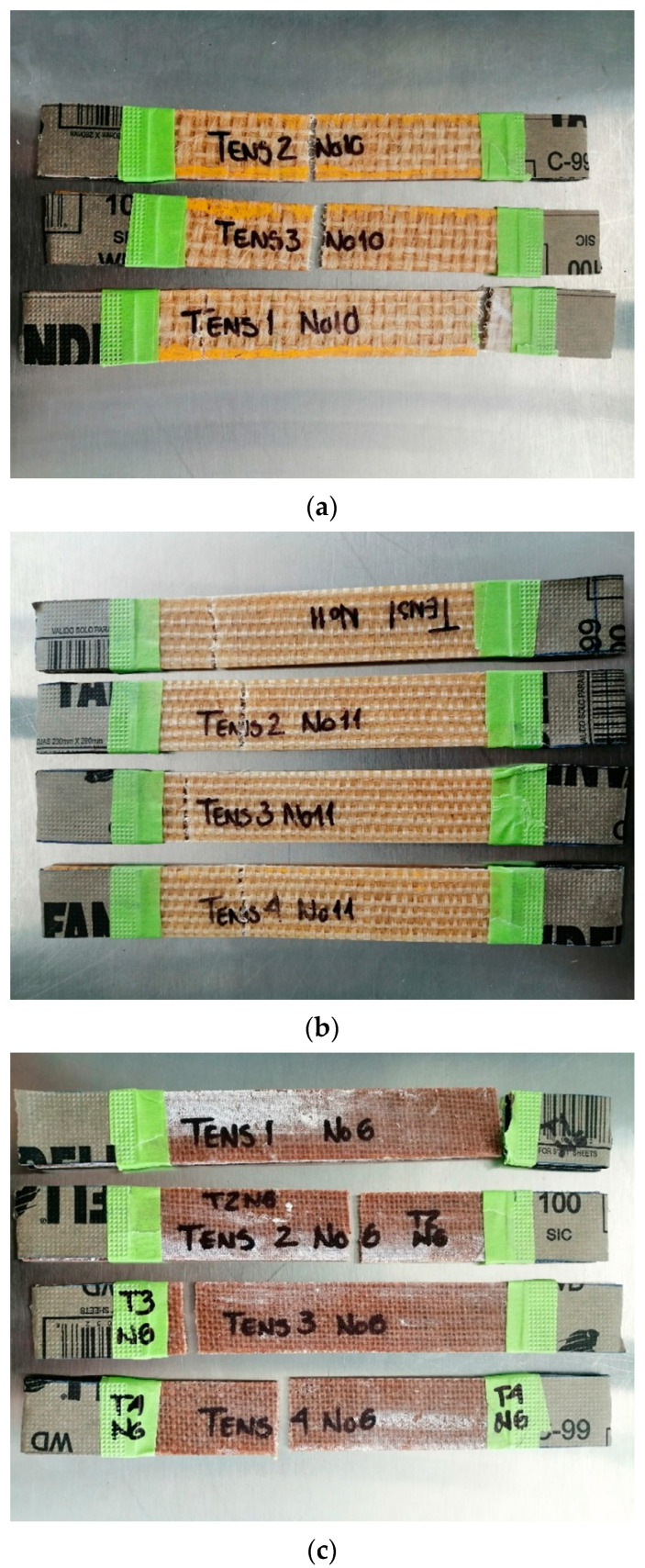
Photographs corresponding to the failure modes in tension for the (**a**) Hen/GP, (**b**) Ixt/GP, (**c**) and Jute/GP biolaminates.

In the second instance, for the natural-fiber-reinforced GP biobased resins (Figure 11), the failure modes are as follows. The Hen/GP biolaminates fail the most by sudden fracture type LGM (Lateral, Gage; Middle) and present type LAT (Lateral, At grip/Tab, Top), as shown in Figure 11a. As GP has a higher molecular structure from plant origin, it anchors reasonably better to the fiber’s microfibrils, gaining resistance. For the Ixt/GP biolaminates, all fail by sudden fracture type LGT (Lateral, Gage, Top), as shown in Figure 11b. It is possible that, as a good bonding is expected between the bioepoxy and the disorderly morphology of the Ixtle tows, the failure occurs where a high-stress concentration is located, in this case, near the grip. Finally, the Jute/GP biolaminates fail all by a different fracture type: LAT (Lateral, At grip/Tab, Top), LGM (Lateral, Gage, Middle), and LGT (Lateral, Gage, Top), as shown in Figure 11c. Independently of the bioepoxy resin, the Jute inner fabric has flaws such as fiber misalignment, fabric distortion, and tow waviness, determined by the multiple failure modes identified. However, as cited in the previous section, Jute has the highest mechanical properties [36,37,38].

### 3.5. Failure Evaluation of Bending Specimens

Similar to the failure explanation in the tensile mode, the failure of biolaminates after the flexural test was also evaluated. This examination was useful to classify their primary failure mode. The deflection typically occurred at the middle of the biolaminates, which was in contact with the machine head, considered to be a loaded edge. The deflection typically occurred in the middle of the laminates, which was in contact with the machine head, therefore considered to be a loaded edge. The loading produces the maximum bending stress by deflecting the laminate and even fracture of the laminate’s outer fibers under excessive loading. In our case, the fibers did not break during the tests.

**Figure 12:**
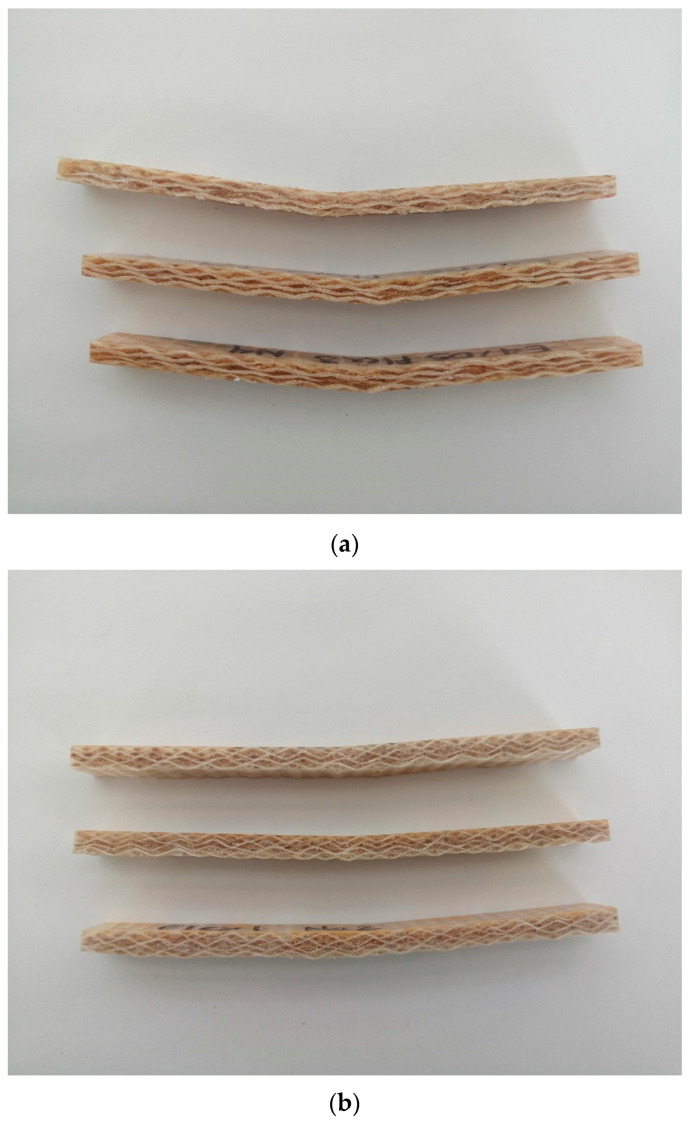

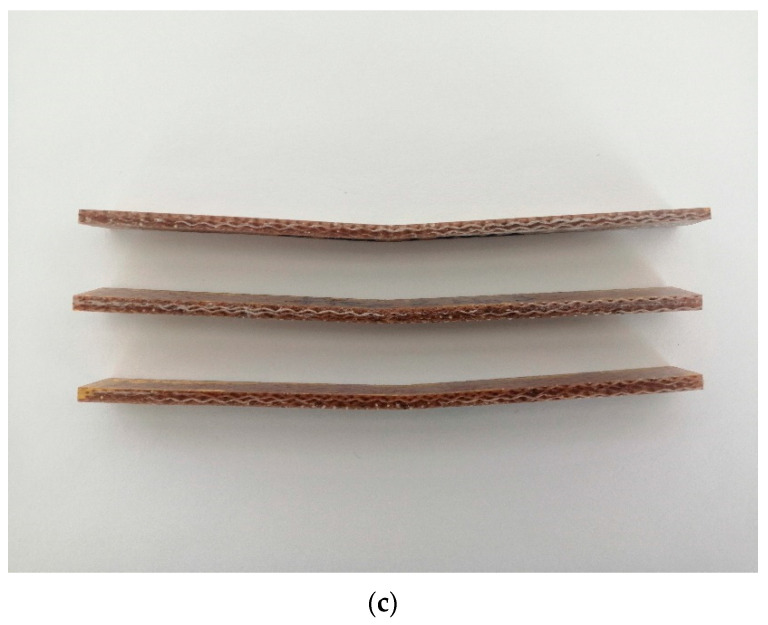
Failure modes of the biolaminates (**a**) Henequen, (**b**) Ixtle, and (**c**) Jute after bending tests.

Regardless of the biobased epoxy resins (EVO and GP), each natural fiber evaluated in this work has its failure mode. All specimens fail by progressive deflection, and separation into two beams was not observed. The Henequen specimens fail by sudden fracture type TAB (Tension, At loading nose, Bottom), as shown in Figure 12a. As can be seen, the Henequen specimens present the largest deflection of the three fibers. It also noted that the Henequen specimens present fiber bifurcation at the loading point.

The Ixtle specimens fail by sudden fracture type TAB (Tension, At loading nose, Bottom), as shown in Figure 12b. It is also remarkable that the resin changed its color, showing localized white marks at the loading point. This type of failure mode makes it possible to find in plain wave fabric with equilibrated wrap and weft tows, such as for Ixtle in this study.

Finally, the Jute specimens fail also by sudden fracture type TAB (Tension, At loading nose, Bottom), as shown in Figure 12c. The deflection is the lowest of the three fibers, commonly found in fabrics with high closure factors. The failure mode is according to the typical codes of ASTM D7264.

## 4. Conclusions

This work reports the use of some relevant natural fibers, such as Henequen, Ixtle, and Jute, as practical reinforcement fabrics for two biobased epoxy resins.

The biolaminates were manufactured using the VARI process, which allowed a balance in the fiber volume fraction and low-cost processing.

The biolaminates were prepared correctly, and the specimens of tension and flexion were cut to proceed with the mechanical experimentation.

Regarding the biobased nature of epoxy resins, the EVO bioresin’s mechanical performance was favored with the Ixtle fiber, followed by the Henequen and Jute fabrics. In the case of the GP natural resin, the best performance was found with the use of Henequen followed by the Ixtle and Jute fibers.

The Jute biolaminates presented the highest stiffness and strength, whereas the Henequen biolaminates showed the largest ultimate strain, and the Ixtle biolaminates offered the best balance for resistance and elongation.

A sudden fracture Lateral, Gage, Top type was observed for all biolaminates. Most specimens failed because the stress concentration was favored by the excellent bonding between the fiber microfibrils and biobased epoxy resins.

The results should drive further applications in industries such as automotive, commodities, construction, and where a balance of low weight, low cost, sustainability, and low carbon footprint is required. As stated in this work, biolaminates overall mechanical properties could afford nonstructural applications where GFRP are used.

## Figures and Tables

**Table 1:** General properties of natural fibers.

Parameter	Henequen	Ixtle	Jute
**Ultimate stress (MPa)**	13.21 ± 2.45	18.34 ± 4.41	45.46 ± 1.34
**Modulus of elasticity (GPa)**	12.92 ± 4.12	26.51 ± 5.65	67.42 ± 3.85
Cellulose content (%) *	60.00–73.00	46.00–48.00	64.40–70.01
Hemicellulose content (%) *	10.00–14.00	17.00–20.00	12.00–13.00
Lignin content (%) *	11.40–19.5	11.00–12.00	11.80–14.10
**Density (g/cm^3^)**	1.12 ± 0.009	1.02 ± 0.003	1.23 ± 0.001

* Data from the literature [25,26,27,28].

**Table 2:** Mechanical properties of biobased resins.

Material	Young Modulus (GPa)	Maximum Strength (MPa)	Elongation (%)	Flexural Modulus (GPa)	Bending Strength (MPa)	Flexural Strain (%)
EVO	3.4	68	6.5	3.2	117	8.5
GP	3.2	50	1.6	3.3	114	4.7

**Table 3:** Volume fractions of EVO and GP biolaminates.

Material	Fiber Fraction (%)	Resin Fraction (%)	Void Fraction (%)
Hen/EVO	24.60	75.30	0.10
Ixt/EVO	16.81	83.10	0.09
Jute/EVO	25.55	74.31	0.14
Hen/GP	24.80	75.11	0.09
Ixt/GP	15.42	84.51	0.07
Jute/GP	29.00	70.90	0.10

## References

[1] Castro A.C.M., Carvalho J., Ribeiro M.C.S., Meixedo J.P., Silva F.J., Fiúza A., Dinis M.D.L. (2014). An integrated recycling approach for GFRP pultrusion wastes: Recycling and reuse assessment into new composite materials using Fuzzy Boolean Nets. J. Clean. Prod..

[2] Aono Y., Murae S., Kubo T. (2011). Static Mechanical Properties of GFRP Laminates with Waste GFRP Interleaf. Procedia Eng..

[3] Ramesh M., Palanikumar K., Reddy K.H. (2013). Comparative Evaluation on Properties of Hybrid Glass Fiber- Sisal/Jute Reinforced Epoxy Composites. Procedia Eng..

[4] Geng Z., Yang S., Zhang L., Huang Z., Pan Q., Li J., Weng J., Bao J., You Z., He Y. (2018). Self-Extinguishing Resin Transfer Molding Composites Using Non-Fire-Retardant Epoxy Resin. Materials.

[5] Mihaich E., Friederich U., Caspers N., Hall A.T., Klecka G.M., Dimond S.S., Staples C.A., Ortego L.S., Hentges S.G. (2009). Acute and chronic toxicity testing of bisphenol A with aquatic invertebrates and plants. Ecotoxicol. Environ. Saf..

[6] Kuo P.-Y., Sain M., Yan N. (2014). Synthesis and characterization of an extractive-based bio-epoxy resin from beetle infested Pinus contorta bark. Green Chem..

[7] Nikafshar S., Zabihi O., Hamidi S., Moradi Y., Barzegar S., Ahmadi M., Naebe M. (2017). A renewable bio-based epoxy resin with improved mechanical performance that can compete with DGEBA. RSC Adv..

[8] Ali A., Shaker K., Nawab Y., Ashraf M., Basit A., Shahid S., Umair M. (2015). Impact of hydrophobic treatment of jute on moisture regain and mechanical properties of composite material. J. Reinf. Plast. Compos..

[9] Koyuncu M., Karahan M., Karahan N., Shaker K., Nawab Y. (2016). Static and Dynamic Mechanical Properties of Cotton/Epoxy Green Composites. Fibres Text. East. Eur..

[10] Rehman M.M., Zeeshan M., Shaker K., Nawab Y. (2019). Effect of micro-crystalline cellulose particles on mechanical properties of alkaline treated jute fabric reinforced green epoxy composite. Cellulose.

[11] Jabbar A., Militký J., Kale B.M., Rwawiire S., Nawab Y., Baheti V. (2016). Modeling and analysis of the creep behavior of jute/green epoxy composites incorporated with chemically treated pulverized nano/micro jute fibers. Ind. Crop. Prod..

[12] Prileschajew N. (1909). Oxydation ungesättigter Verbindungen mittels organischer Superoxyde. Ber. Dtsch. Chem. Ges..

[13] Jawaid M., Khalil H.A., Hassan A., Dungani R., Hadiyane A. (2013). Effect of jute fibre loading on tensile and dynamic mechanical properties of oil palm epoxy composites. Compos. Part B Eng..

[14] Salman S.D. (2019). Effects of jute fibre content on the mechanical and dynamic mechanical properties of the composites in structural applications. Def. Technol..

[15] Cicala G., Pergolizzi E., Piscopo F., Carbone D.C., Recca G. (2018). Hybrid composites manufactured by resin infusion with a fully recyclable bioepoxy resin. Compos. Part B Eng..

[16] Li W., Krehl J., Gillespie J.W., Heider D., Endrulat M., Hochrein K., Dunham M.G., Dubois C.J. (2004). Process and Performance Evaluation of the Vacuum-Assisted Process. J. Compos. Mater..

[17] Hsiao K.-T., Heider D. (2012). Vacuum assisted resin transfer molding (VARTM) in polymer matrix composites. Manuf. Tech. Polym. Matrix Compos..

[18] Puglia D., Biagiotti J., Kenny J.M. (2005). A Review on Natural Fibre-Based Composites—Part II. J. Nat. Fibers.

[19] Sreenivasan S., Sulaiman S., Ariffin M.K.A.M., Baharudin B.H.T., Khalina A. (2018). Physical Properties of Novel Kenaf Short Fiber Reinforced Bulk Molding Compounds (BMC) For Compression Moulding. Mater. Today Proc..

[20] Holmes M. (2017). Aerospace looks to composites for solutions. Reinf. Plast..

[21] González C., Vilatela J., Molina-Aldareguía J., Lopes C., Llorca J. (2017). Structural composites for multifunctional applications: Current challenges and future trends. Prog. Mater. Sci..

[22] Hossain R., Islam A., Van Vuurea A., Verpoest I. (2013). Tensile Behavior of Environment Friendly Jute Epoxy Laminated Composite. Procedia Eng..

[23] Hachemane B., Zitoune R., Bezzazi B., Bouvet C. (2013). Sandwich composites impact and indentation behaviour study. Compos. Part B Eng..

[24] Akil H., Omar M., Mazuki A., Safiee S., Ishak Z., Abu Bakar A., Akil H.M., Omar M.F., Mazuki A.A.M., Safiee S. (2011). Kenaf fiber reinforced composites: A review. Mater. Des..

[25] Lim J.K., Raja V.S., Shoji T.B.T.-S.C.C. (2011). Stress corrosion cracking (SCC) in polymer composites. Stress Corrosion Cracking.

[26] Chand N., Fahim M.B.T.-T. (2008). Natural fibers and their Composites. Woodhead Publishing Series in Composites Science and Engineering.

[27] Márquez A., Cazaurang N., González I., Colunga-GarciaMarin P. (1996). Extraction of chemical cellulose from the fibers of Agave lechuguilla Torr. Econ. Bot..

[28] Hernandez C., Rosa D. (2016). Extraction of cellulose nanowhiskers: Natural fibers source, methodology and application. Polymer Science: Research Advances, Practical Applicational and Educational Aspects.

[29] Adhikari R.K., Gowda B.K. (2017). Exploration of mechanical properties of banana/jute hybrid polyester composite. Mater. Today Proc..

[30] Gon D., Das K., Paul P., Maity S. (2013). Jute Composites as Wood Substitute. Int. J. Text. Sci..

[31] Jawaid M., Khalil H.A., Bakar A.A. (2011). Woven hybrid composites: Tensile and flexural properties of oil palm-woven jute fibres based epoxy composites. Mater. Sci. Eng. A.

[32] Mishra V., Biswas S. (2013). Physical and Mechanical Properties of Bi-directional Jute Fiber Epoxy Composites. Procedia Eng..

[33] Rong M.Z., Zhang M.Q., Liu Y., Yang G.C., Zeng H.M. (2001). The effect of fiber treatment on the mechanical properties of unidirectional sisal-reinforced epoxy composites. Compos. Sci. Technol..

[34] Padmavathi T., Naidu S.V., Rao R. (2012). Studies on Mechanical Behavior of Surface Modified Sisal Fibre–Epoxy Composites. J. Reinf. Plast. Compos..

[35] Gupta M., Srivastava R. (2016). Properties of sisal fibre reinforced epoxy composite. Indian J. Fibre Text. Res..

[36] Gupta M., Srivastava R. (2014). Tensile and Flexural Properties of Sisal Fibre Reinforced Epoxy Composite: A Comparison between Unidirectional and Mat form of Fibres. Procedia Mater. Sci..

[37] Chokshi S., Gohil P., Patel D. (2020). Experimental investigations of bamboo, cotton and viscose rayon fiber reinforced Unidirectional composites. Mater. Today Proc..

[38] Elanchezhian C., Ramnath B.V., Ramakrishnan G., Rajendrakumar M., Naveenkumar V., Saravanakumar M. (2018). Review on mechanical properties of natural fiber composites. Mater. Today Proc..

